# Donor-Derived Cell-Free DNA in Pancreas-Kidney, Heart-Kidney, and Liver-Kidney Multiorgan Transplant Recipients (MOTR)

**DOI:** 10.3389/ti.2025.15823

**Published:** 2026-01-19

**Authors:** Gaurav Gupta, David Wojciechowski, Alp Sener, Timothy Gong, Ty B. Dunn, Nadiesda Costa, Jon S. Odorico, Reem Daloul, Vinayak S. Rohan, D. Giovanni Biagini, David Barnes, Navchetan Kaur, Geethanjali Gude, Ebad Ahmed, Jing Xie, Catherine J. Spellicy, Nour Al Haj Baddar, Michelle S. Bloom, Zachary Demko, Adam Prewett, Phil Gauthier, Sangeeta Bhorade, Hossein Tabriziani, Sanjeev K. Akkina

**Affiliations:** 1 Virginia Commonwealth University, Richmond, VA, United States; 2 UT Southwestern Medical Center, Dallas, TX, United States; 3 Western University, Schulich School of Medicine and Dentistry, London, ON, Canada; 4 Baylor University Medical Center, Dallas, TX, United States; 5 Perelman School of Medicine, University of Pennsylvania, Philadelphia, PA, United States; 6 MedStar Georgetown University Hospital, Washington, DC, United States; 7 Medical College of Wisconsin, Milwaukee, WI, United States; 8 The Ohio State University, Columbus, OH, United States; 9 Northwestern University Feinberg School of Medicine, Chicago, IL, United States; 10 Natera, Inc., Austin, TX, United States; 11 Loyola University Medical Center, Maywood, IL, United States

**Keywords:** allograft rejection, dd-cfDNA, heart transplant, kidney translant, multi-organ transplant

## Abstract

Donor-derived cell free DNA (dd-cfDNA) is an established biomarker for detection of rejection in single organ transplants; data is limited in multi-organ transplant (MOT) recipients. “Use of dd-cfDNA in Multi-Organ Transplant Recipients” (MOTR) was a multicenter, prospective, cross-sectional study that assessed dd-cfDNA fraction (%) and donor quantity score (DQS, cp/mL) in pancreas-kidney (PKT), heart-kidney (HKT), and liver-kidney (LKT) recipients. We explored dd-cfDNA baseline levels across the different organ combinations, and compared them to kidney-only (KT) and heart-only (HT) transplant recipients. Among 347 MOT recipients from 18 sites (PKT = 183, HKT = 57, LKT = 107), most (88.2%) had simultaneous transplants. Median dd-cfDNA levels in PKT and HKT recipients were not significantly different from KT; median dd-cfDNA levels among HKT recipients were significantly higher than in HT recipients (p < 0.001). In LKT recipients, median dd-cfDNA was significantly higher compared to KT (p < 0.001). dd-cfDNA showed associations with organ impairment indicated by abnormal values of pancreatic and liver enzymes in PKT and LKT. As the largest multi-center study to date evaluating dd-cfDNA levels in MOT recipients, MOTR showed that organ-specific physiology affects dd-cfDNA levels across organ transplant combinations, laying the foundation for future efforts to use dd-cfDNA to assess organ-specific signatures of allograft injury in MOT recipients.

## Graphical Abstract

**Figure:**
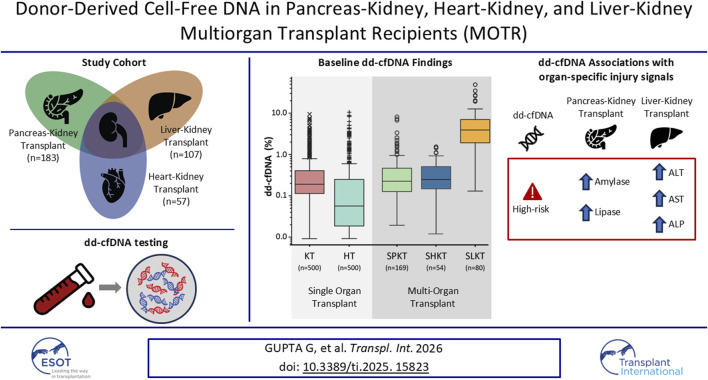

## Introduction

Multi-organ transplantation (MOT) is an increasingly important therapeutic strategy driven by the rising burden of chronic disease, aging patient population, and the complex interplay of multi-organ dysfunction. As more patients present with concurrent multiple organ failure, MOT is the optimal treatment, providing life-saving intervention [1, 2]. The number of patients undergoing MOT between 2011 and 2021 with a kidney and additional solid organ has grown 23.4% [3]. Clinical management of MOT recipients is significantly more complex than single organ transplant, requiring more resources for monitoring and coordination between multiple clinical teams. Thus, there is a growing need to understand the applicability of emerging non-invasive biomarkers in this expanding high-risk population.

The physiological rationale for MOT is well-established across different clinical scenarios. Simultaneous pancreas-kidney transplant (SPKT) improves outcomes in patients with diabetes and end-stage renal disease (ESRD) [4]. Heart-kidney transplantation (HKT) benefits patients with coexisting cardiac and renal failure, a common clinical scenario resulting from the inter-independence of both organs [5], reducing the risk of calcineurin inhibitor (CNI)-induced nephrotoxicity during heart transplantation which has contributed to a six-fold rise in US transplants since 2010 [6]. Simultaneous liver-kidney transplantation (SLKT) treats end-stage liver disease with concomitant kidney impairment or ESRD [7, 8], a common complication in this population [9]. Also, patients who may not qualify for a kidney transplant alone due to significant cardiac or hepatic dysfunction may qualify through the addition of the heart or liver allograft.

Traditional biomarkers for monitoring acute rejection (AR) in MOT are limited in their prognostic utility. For example, serum creatinine (sCr), proteinuria, amylase, lipase, and liver enzymes (like alanine aminotransferase (ALT), alkaline phosphatase (ALP), and aspartate aminotransferase (AST)) are non-specific and can be confounded by unrelated factors [10–12]. Heart transplant biomarkers like B-type natriuretic (BNP) and N-terminal pro-BNP (NT-proBNP) are weakly-associated with heart transplant rejection [13]. Although biopsy remains the gold standard, its invasive nature and susceptibility to sampling error highlight the need for non-invasive biomarkers with improved sensitivity and specificity for organ injury in patients with MOT.

Donor-derived cell-free DNA (dd-cfDNA) is a validated biomarker for detecting AR in kidney [14, 15], lung [16], and heart single-organ transplant recipients [17]. Numerous studies have consistently demonstrated the high negative predictive value and strong correlation of dd-cfDNA with molecular and histologic rejection phenotypes across single-organ transplant types [18–22]. While dd-cfDNA was historically reported as a fraction of total circulating cf-DNA, studies have demonstrated that quantification of the plasma concentration of dd-cfDNA improves discrimination of rejection and reduces confounding from fluctuations in total cfDNA [23, 24]. Moreover, the combination of dd-cfDNA fraction and a donor quantity score (DQS), which estimates the quantity of dd-cfDNA in copies per mL of patient’s plasma, has also been shown to improve accuracy in the detection of AR with rejection thresholds of ≥1.0% and/or ≥78 cp/mL in kidney [25, 26], and ≥0.26% and/or ≥18 cp/mL in heart transplants [27]. While dd-cfDNA has been studied and characterized in SPKT recipients in a single-center study [28], its behavior in other MOT combinations remains largely unexplored, highlighting the need for additional research.

Here we introduce the “Use of dd-cfDNA in Multi-Organ Transplant Recipients” (MOTR) study; a multicenter, prospective, cross-sectional study designed to evaluate the dd-cfDNA baseline levels in real-world cohort of MOT recipients who were either clinically stable or who underwent a scheduled biopsy of at least one transplanted organ. This study analyzed dd-cfDNA levels, as a fraction (%), and donor quantity score (DQS; copies/mL) in pancreas-kidney (PK), heart-kidney (HK), and liver-kidney (LK) transplant recipients, and compared them to matched, real-world single-organ KT and HT recipients.

## Materials and Methods

### Study Design

The MOTR study was an international, prospective, cross-sectional, multicenter study that evaluated dd-cfDNA baseline levels in patients with MOT. The studies involving human participants were reviewed and approved by local or central institutional review boards (IRBs, Supplementary Table S1). All PK, HK and LK transplant recipients who met the eligibility criteria were consented according to the IRB-approved protocol. The study has been performed in full adherence to the Declaration of Helsinki.

Patients were considered eligible for enrollment into the study if they met the following inclusion criteria: (1) age ≥18 years at the time of signing informed consent, (2) history of pancreas-kidney, kidney-heart, or liver-kidney transplant, (3) either considered clinically stable (defined as no history of rejection or no clinical indication of rejection within 3 months prior to enrollment in the study), or scheduled to undergo a biopsy of at least one of the transplanted organs, (4) able to read, understand, and provide written informed consent, and (5) willing and able to comply with the study-related procedures. Patients were excluded from this study if, at the time of enrollment they (1) were pregnant, (2) were on dialysis, (3) had received an allograft from an identical twin, and/or (4) had more than 2 unique organ transplant types (i.e.: pancreas, liver, heart).

Clinical and biological data related to recipient characteristics, donor characteristics, transplant procedure, and contemporaneous laboratory tests including SCr, lipase, amylase, ALP, AST, ALT, DSA, and viral loads, were collected for patients in both the stable arm and the biopsy-matched arm (Table 1).

**TABLE 1:** Demographics and clinical characteristics of MOTR study patients.

Recipient characteristics	All patients (N = 347)	Pancreas-kidney Cohort (N = 183)	Heart-kidney cohort (N = 57)	Liver-kidney (N = 107)	P value^a^	Pairwise p-values^b^ PKT-HKT PKT-LKT HKT-LKT
Age (years)	57.1 (48.7–65.9)	51.1 (45.4–57.3)	62.1 (54.6–68.9)	64.8 (59.3–71.1)	<0.0001	<0.0001 <0.0001 0.2901
Sex ratio Male Female	224 (64.6%) 123 (35.4%)	115 (62.8%) 68 (37.2%)	45 (78.9%) 12 (21.1%)	64 (59.8%) 43 (40.2%)	0.0398	0.1094 1.0000 0.0647
BMI (kg/m^2^)	28.2 (24.9–32.7)	27.2 (24.1–31.8)	28.9 (26.0–32.9)	29.0 (26.0–33.4)	0.0404	0.1958 0.0828 1.0000
Time from most recent transplant to dd-cfDNA testing (months)	23.5 (8.4–52.7)	33.8 (9.2–73.9)	13.1 (6.4–33.2)	19.0 (8.2–41.8)	0.0001	0.0003 0.0220 0.2001
Race White African American Other Asian Unknown	179 (51.6%) 96 (27.7%) 6 (1.7%) 11 (3.2%) 55 (15.9%)	82 (44.8%) 44 (24.0%) 4 (2.2%) 3 (1.6%) 50 (27.3%)	18 (31.6%) 32 (56.1%) 0 (0.0%) 5 (8.8%) 2 (3.5%)	79 (73.8%) 20 (18.7%) 2 (1.9%) 3 (2.8%) 3 (2.8%)	<0.0001	<0.0001 <0.0001 <0.0001
Ethnicity Not hispanic or latino Hispanic or latino Unknown	263 (75.8%) 28 (8.1%) 56 (16.1%)	116 (63.4%) 16 (8.7%) 51 (27.9%)	53 (93.0%) 3 (5.3%) 1 (1.8%)	94 (87.9%) 9 (8.4%) 4 (3.7%)	<0.0001	0.0002 <0.0001 1.000
Contemporaneous DSA status to dd-cfDNA testing Yes No	23 324	12 171	7 50	4 103	0.1115	0.7925 1.0000 0.2379
Transplant sequence Non-simultaneous Simultaneous Repeat	36 (10.4%) 303 (87.3%) 8 (2.3%)	7 (3.8%) 169 (92.3%) 7 (3.8%)	3 (5.3%) 52 (91.2%) 2 (3.5%)	26 (24.3%) 80 (74.8%) 1 (0.9%)	<0.0001	0.8934 <0.0001 0.00181
Biopsies * For-cause* *For-protocol* Not reported	49 (14.1%) 48 (13.8%) 250 (72.0%)	34 (18.6%) 6 (3.3%) 143 (78.1%)	7 (12.3%) 27 (47.4%) 23 (40.4%)	8 (7.5%) 15 (14.0%) 84 (78.5%)	<0.0001	<0.0001 0.0008 <0.0001
Mortality status Alive Died	345 (99.4%) 2 (0.6%)	182 (99.5%) 1 (0.5%)	57 (100.0%) 0 (0.0%)	106 (99.1%) 1 (0.9%)	0.7509	1.0000 1.0000 1.0000
Kidney donor characteristics						
Donor age (years)	32.0 (22.0–41.0)	27.0 (20.0–35.0)	33.0 (27.0–45.0)	40.0 (30.2–49.0)	<0.0001	0.0009 <0.0001 0.0768
Donor relationship to recipient Not related Biologically related	342 (98.6%) 5 (1.4%)	178 (97.3%) 5 (2.7%)	57 (100.0%) 0 (0.0%)	107 (100.0%) 0 (0.0%)	0.1030	1.0000 0.6259 1.0000
Donor type Living DCD DBD Not reported	11 (3.2%) 43 (12.4%) 270 (77.8%) 23 (6.6%)	7 (3.8%) 22 (12.0%) 144 (78.7%) 10 (5.5%)	3 (5.3%) 2 (3.5%) 47 (82.5%) 5 (8.7%)	1 (0.9%) 19 (17.8%) 79 (73.8%) 8 (7.5%)	0.1190	0.7209 0.7031 0.0805
KDPI	23.0 (6.0–41.0)	11.0 (0.0–26.0)	28.0 (15.2–46.5)	39.0 (24.0–64.0)	<0.0001	<0.0001 <0.0001 0.0424
Pancreas/Heart/Liver donor characteristics (non-simultaneous)						
Donor age (years)	28.0 (20.5–39.5)	20.0 (17.5–24.5)	29.0 (24.8–32.5)	33.0 (25.5–45.0)	0.0090	0.6017 0.0088 1.0000
Donor type DCD DBD Not reported	3 (6.5%) 36 (78.3%) 7 (15.2%)	1 (7.1%) 7 (50.0%) 6 (42.9%)	0 (0.0%) 5 (100.0%) 0 (0.0%)	2 (7.4%) 24 (88.9%) 1 (3.7%)	0.0132	​

Categorical variables are shown as median (IQR). BMI: body mass index, dd-cfDNA: Donor-derived cell-free DNA, DSA: donor specific antibodies, CPRA: calculated panel reactive antibody, DCD: donation after cardiac death, DBD: donation after brain death, KDPI: kidney donor profile index, CMV: cytomegalovirus, EBV: Epstein-Barr virus, HIV: Human immunodeficiency virus.

^a^ Kruskal-Wallis test comparing PKT, HKT, and LKT, cohorts.

^b^ Post-hoc Mann-Whitney U or Chi-Square test comparing PKT-HKT, PKT-LKT, and HKT-LKT, with Bonferroni correction for significant Kruskal-Wallis values.

### dd-cfDNA Testing

dd-cfDNA samples were analyzed using the methodology previously described (the Prospera™ test; Natera, Inc., Austin, TX) [29]. Blood samples for dd-cfDNA testing were collected in two 10 mL Streck Cell-Free DNA BCT tubes and shipped at room temperature to Natera’s Clinical Laboratory Improvement Amendments-certified and College of American Pathologists-accredited laboratory. Samples were processed within 8 days of collection, or according to previously validated standard operating protocol [30]. The algorithm was designed to provide a single composite value by combining non-recipient cfDNA for both dd-cfDNA % and DQS, which is an estimate of the concentration of dd-cfDNA in plasma reported as genomic copies per mL (cp/mL). Blood draws for dd-cfDNA testing were performed within 0–7 days before biopsy in the biopsy-matched arm, or according to clinical standard in the stable arm. Results of the investigational test (dd-cfDNA) were not shared with the patients or the attending physicians, and thus not used in patient management.

### Biopsy Classification

Allograft biopsies of kidney, pancreas, heart, and liver transplants were graded as previously reported [31–34]. Biopsy reports were centrally reviewed by pathologists for data accuracy.

### Statistical Analysis

dd-cfDNA levels in PKT, HKT, LKT recipients were compared to real-world cohorts of KT and HT recipients matched for time post-transplant.

Statistical analyses were performed in Python (3.8.10). Normally distributed variables were reported as mean and standard deviation; non-normally distributed variables were reported as median and interquartile range (IQR). Categorical variables were represented as counts and percentages and evaluated using Chi-square test as appropriate. Kruskal-Wallis tests were performed for groupwise comparisons. For significant p-values, post-hoc tests of chi-square or Mann Whitney U tests were performed, as appropriate, with Bonferroni corrections for multiple testing.

Random forest classifiers were applied to identify the relationship between known clinical variables and positivity of dd-cfDNA (i.e., high risk by dd-cfDNA) testing. dd-cfDNA positivity was defined using the clinical thresholds established for kidney-alone transplant rejection (dd-cfDNA%≥1.0% OR DQS≥78.0 cp/mL) for the PKT and HKT cohorts, and an investigational, unvalidated threshold for liver-alone transplant rejection (dd-cfDNA%≥10.0%) based on prior studies [35–37] for the LKT cohort. Modeling was not feasible in the HKT cohort due to the limited number of dd-cfDNA-positive cases. Modeling included PKT cases with complete known matched eGFR (estimated glomerular filtration rate), amylase and lipase values, and LKT cases with complete known matched eGFR, ALP, AST, and ALT values, and was trained on these values along with time from transplant, Kidney Donor Profile Index (KDPI), and body mass index (BMI) and optimized for F1 score. The Shapley values of these features were assessed on a holdout random set of 20% of the complete cases.

Spearman correlation analyses were performed between dd-cfDNA (%, cp/mL) and the clinical biomarkers relevant to each transplanted organ, as described above. For each variable pair, correlation was calculated using cases with complete data.

### SRTR Database

This study used data from the Scientific Registry of Transplant Recipients (SRTR). The SRTR data system includes data on all donor, wait-listed candidates, and transplant recipients in the US, submitted by the members of the Organ Procurement and Transplantation Network (OPTN). The Health Resources and Services Administration (HRSA), U.S. Department of Health and Human Services provides oversight to the activities of the OPTN and SRTR contractors. Contemporaneous data from the Scientific Registry of Transplant Recipients (SRTR) was assessed for comparison.

The data reported here have been supplied by the Hennepin Healthcare Research Institute (HHRI) as the contractor for SRTR. The interpretation and reporting of these data are the responsibility of the author(s) and in no way should be seen as an official policy of or interpretation by the SRTR or the U.S. Government.

Contemporaneous data from SRTR were queried to compare the MOTR study cohorts (PKT, HKT, and LKT) to the corresponding national cohorts for each MOT type, to assess representativeness and generalizability. Variables included MOT recipient age, sex, BMI, race/Ethnicity, and repeat transplant status as indicated in Supplementary Table S2.

## Results

### Study Cohort

**FIGURE 1:**
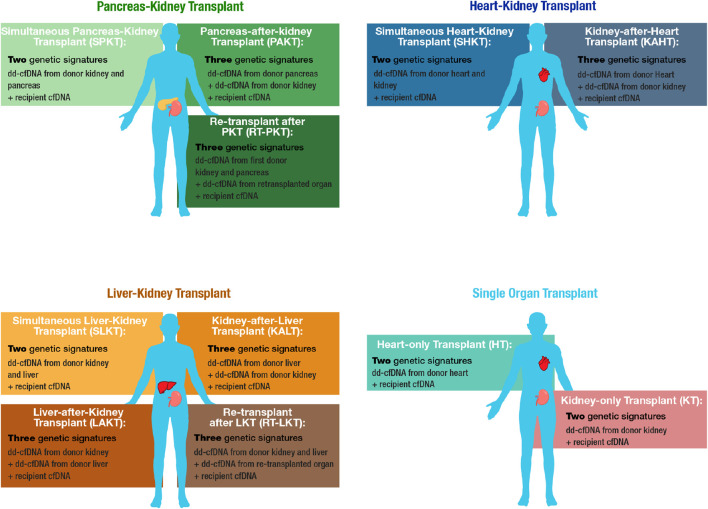
Overview of MOT cohorts and corresponding cfDNA constituents. MOT cohorts include Pancreas-Kidney transplant (PKT), Heart-Kidney transplant (HKT), and Liver-Kidney transplant (LKT). Matched-single-organ transplant cohorts, kidney-only transplant (KT) and heart-only transplant (HT), serve as comparators.

**FIGURE 2:**
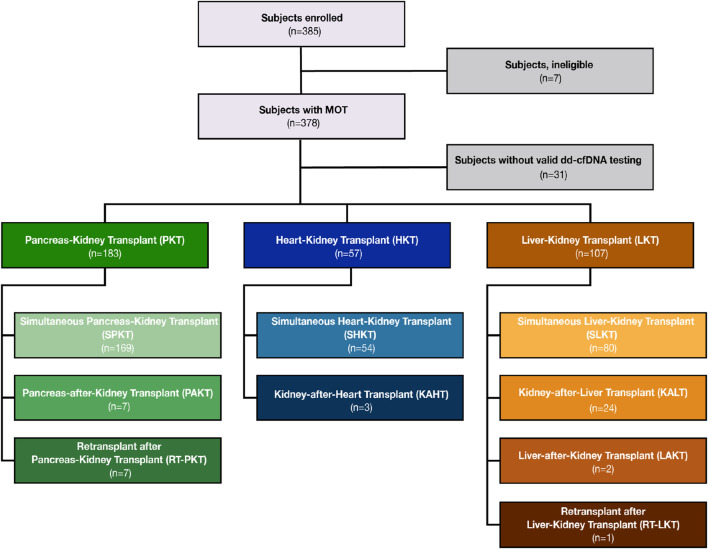
CONSORT diagram of study enrollment and breakdown of organ groups by transplant sequence.

Between October 2020 and June 2022, 385 subjects from 18 sites were enrolled in the MOTR Study (Supplementary Table S1). Of these, 38 subjects were excluded due to ineligibility or lack of valid dd-cfDNA testing (e.g., insufficient plasma volume). Of the remaining 347 transplant recipients, 183 had PKT, including 169 with a simultaneous pancreas-kidney transplant (SPKT; 92.4%), 7 with a pancreas-after-kidney transplant (PAKT; 3.8%). 7 patients (3.8%) underwent repeat transplant following a kidney-pancreas transplant: Four received kidney re-transplant after SPK, and three received a pancreas retransplant (one after PAK, two after SPK). 57 subjects had HKT, including 54 with a simultaneous heart-kidney transplant (SHKT; 94.7%) and 3 with a kidney-after-heart transplant (KAHT; 5.3%). 107 subjects had LKT including 80 with a simultaneous kidney-liver transplant (SLKT; 74.8%), 24 with a kidney-after-liver transplant (KALT; 22.4%), two with a liver-after-kidney transplant (LAKT; 1.9%), and one (0.9%) with a liver re-transplant following a kidney-after-liver transplant (Figures 1, 2).

The MOTR cohort was 64.6% male (n = 224), had a median age of 57.1 y (IQR: 48.7–65.9), and a median BMI of 28.2 kg/m^2^ (IQR: 24.9–32.7). Patients were 51.6% White, 27.7% African American, and 8.1% Hispanic and/or Latino. In the PKT, HKT, and LKT cohorts, 3.8% (7/183), 5.3% (3/57), and 0.9% (1/107) of the transplanted kidneys were donated by living donors, respectively. The median KDPI across all deceased donor’s kidney transplants was 23.0 (IQR: 6.0–41.0). The majority (72%) of MOTR participants were considered clinically stable at enrollment, and the remaining 28% underwent biopsy; (*for-cause:* n = 49, 50.5%; *for-protocol:* n = 48, 49.5%) (Table 1). *For-cause* and *for*-*protocol* biopsies were observed in 18.6% and 3.3% of PKT, 12.3% and 47.4% of HKT, and 7.5% and 14.0% of LKT recipients, respectively; 70% of all biopsies were performed on the transplanted kidney.

The covariates of the PKT, HKT, and LKT subcohorts, such as MOT recipient demographics including age, sex, and ethnicity, were also compared to a contemporaneous national cohort’s covariates as represented in data requested from the SRTR (https://www.srtr.org/) (Supplementary Table S2).

### dd-cfDNA Fraction and DQS Estimates

In this analysis, a single dd-cfDNA test from each patient was used, along with a matched biopsy, when available. The median time from most recent transplant to dd-cfDNA testing was 23.5 months (IQR: 8.4–52.7) and was significantly longer in the PKT cohort (33.8 months) compared to the HKT (13.1 months) and LKT (19.0 months) cohorts (p < 0.05).

**FIGURE 3:**
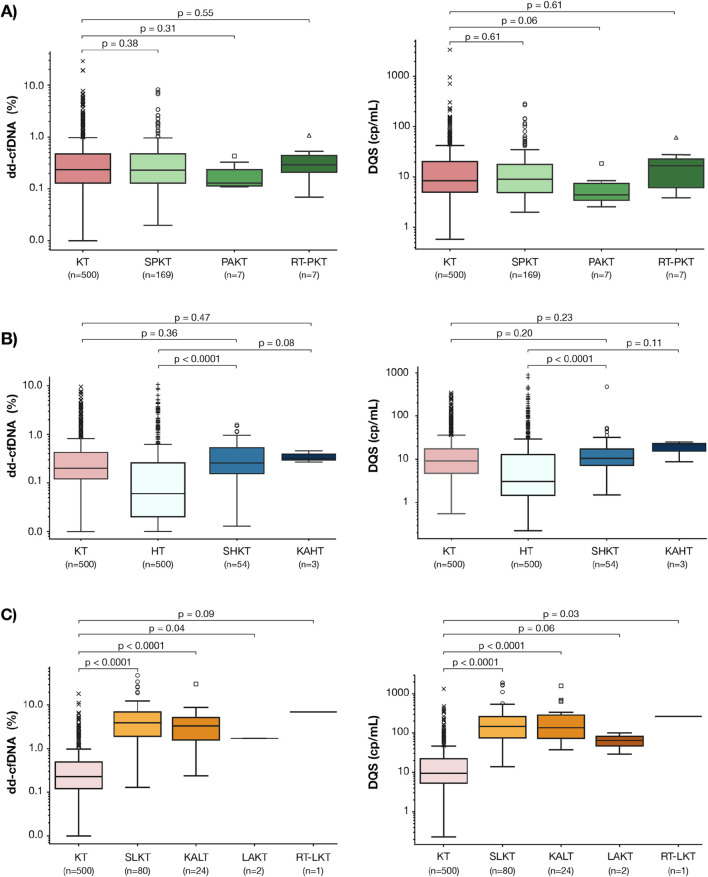
Baseline dd-cfDNA fraction (%) and DQS (cp/mL) levels in the PKT **(A)**, HKT **(B)**, and LKT **(C)** sub-cohorts. Box-and-whisker plots display the median and interquartile range (IQR); whiskers extend to 1.5x IQR representing the expected data range; outliers beyond this range are shown as individual points. P values denote pairwise comparisons in dd-cfDNA levels between: **(A)** SPKT, PAKT, and PK-RT and KT cohorts, **(B)** SHKT, KAHT and KT, HT cohorts, **(C)** SLKT, KALT, LAKT, KL-RT and KT cohorts. PKT: Kidney-Pancreas transplant, SPKT: Simultaneous Kidney-Pancreas transplant, PAKT: Pancreas-after-Kidney transplant, PK-RT: Pancreas-Kidney Repeat transplant, KT: Kidney transplant, HT: Heart transplant, HKT: Kidney-Heart transplant, KAHT: Kidney-after-Heart transplant, SLKT: Simultaneous kidney-liver transplant, KALT: Kidney-after-liver transplant, LAKT: Liver-after-kidney transplant, KL-RT: Kidney-liver Repeat transplant.

Among the PKT recipients, there were no significant differences in the median (IQR) dd-cfDNA % or DQS between the SPKT (dd-cfDNA%: 0.23% (0.13–0.47); DQS: 9 (4–17) cp/mL), PAKT (dd-cfDNA%: 0.13% (0.11–0.23); DQS: 4 (3–7) cp/mL) or those who underwent kidney or pancreas retransplant after PKT (dd-cfDNA%: 0.29% (0.21–0.44); DQS: 16 (6–22) cp/mL) cohorts and the KT cohort (dd-cfDNA%: 0.23% (0.12–0.48); DQS: 9 (5–20) cp/mL) (p = 0.38, 0.31, and 0.55, respectively) (Figure 3A). The distribution of total cfDNA paralleled that of both dd-cfDNA (%) and DQS (cp/mL) among these groups (Supplementary Figure S1A).

Similarly, among the HKT recipients, there were no significant differences in the median (IQR) dd-cfDNA % or DQS (cp/mL) between the SHKT (dd-cfDNA%: 0.21% (0.12–0.42); DQS: 10 (7–18) cp/mL) or the KAHT cohorts (dd-cfDNA%: 0.32% (0.30–0.39; DQS: 22 (15–23) cp/mL) and the KT cohort (dd-cfDNA%: 0.24% (0.12–0.51); DQS: 10 (5–22) cp/mL (p = 0.36, 0.47, respectively) (Figure 3B). Both the median dd-cfDNA % and DQS (cp/mL) were significantly higher among the SHKT cohort when compared to the HT cohort (dd-cfDNA%: 0.21% (0.12–0.42); DQS: 10 (7–18) cp/mL) vs. (dd-cfDNA%: 0.06 (0.02–0.21), DQS: 2 (1–11) cp/mL, p < 0.001) (Figure 3B). There were no differences in total cfDNA when comparing SHKT and KAHT to KT and SHKT and KAHT to HT cohorts (Supplementary Figure S1B).

Among the LKT recipients, both the median (IQR) dd-cfDNA % and DQS for the SLKT (dd-cfDNA%: 3.92% (1.92–6.99); DQS: 147 (75–258) cp/mL) and KALT cohorts (dd-cfDNA%: 3.35% (1.59–5.14); DQS: 35 (73–284) cp/mL) were significantly higher than the KT (dd-cfDNA%: 0.23% (0.12–0.49); DQS: 9 (5–22) cp/mL; p < 0.001 for both) (Figure 3C). Lower total cfDNA levels in the SLKT compared to the KALT cohort may contribute to differences between dd-cfDNA% and DQS (cp/mL) observed between them (Supplementary Figure S1C).

### Patients With Biopsy-Proven Acute Rejection (BPAR)

While this study did not contain sufficient patients with BPAR to assess appropriate dd-cfDNA cutoffs for rejection risk in MOT, we felt it was still illustrative to interpret the data in the context of the validated kidney-only threshold, as we would expect the dd-cfDNA values for kidney-only transplant patients to approximate a lower-bound for the appropriate MOT rejection risk threshold. A total of three patients in the SPKT cohort had kidney BPAR, two of which had dd-cfDNA above the 1.0% threshold used to define increased rejection risk in KT recipients (Supplementary Figure S2A): one recipient with T-cell-mediated rejection (TCMR) had dd-cfDNA levels of 3.11% and 63 cp/mL, while two patients with chronic active antibody-mediated rejection (AMBR) had dd-cfDNA% of 0.51% and 1.59% and DQS of 21 and 55 cp/mL. Three patients among the LKT cohort had BPAR in the kidneys (Supplementary Figure S2B), including TCMR 1A with dd-cfDNA of 1.32%, 86 cp/mL, chronic active ABMR (2.31%, 114 cp/mL), and ABMR (6.98%, 698 cp/mL). No patients in the HKT cohort showed acute rejection on biopsy (Supplementary Figure S2B). Of the remaining 91 biopsies, 68 showed no rejection, 18 showed other findings not representing rejection, and 5 consisted of limited sample tissue and were thus unable to be diagnosed.

### Patients With dd-cfDNA Values Above Thresholds for Kidney-Only Transplant Rejection

Of the 240 non-rejecting PKT and HKT recipients, 21 (19 PKT and 2 HKT) exceeded KT dd-cfDNA thresholds (≥1.0% and/or ≥78 copies/mL). All but one of these transplants were simultaneous; the remaining one was a kidney re-transplant after SPK. Seven patients (33.%) underwent biopsies (6 kidney, 1 heart), all without significant findings. The remaining 14 patients (13 PKT, 1 HKT) were considered clinically stable and did not undergo biopsy; one of these had substantially elevated amylase levels, while the other 13 were clinically stable by all parameters apart from dd-cfDNA. Of the 219 patients with dd-cfDNA below KT threshold, only one experienced BPAR (chronic-active ABMR).

Of the 107 subjects in the LKT cohort, 12 (11.2%; 11 SLKT, 1 KALT) had dd-cfDNA above 10%, which prior studies have posited as an appropriate threshold for rejection in liver-only transplants [35–37]. Two of these subjects received surveillance biopsies of the kidney, both demonstrating no rejection; no patients received biopsies of the liver.

### Variables Associated With Elevated dd-cfDNA Levels in MOT Recipients

**FIGURE 4:**
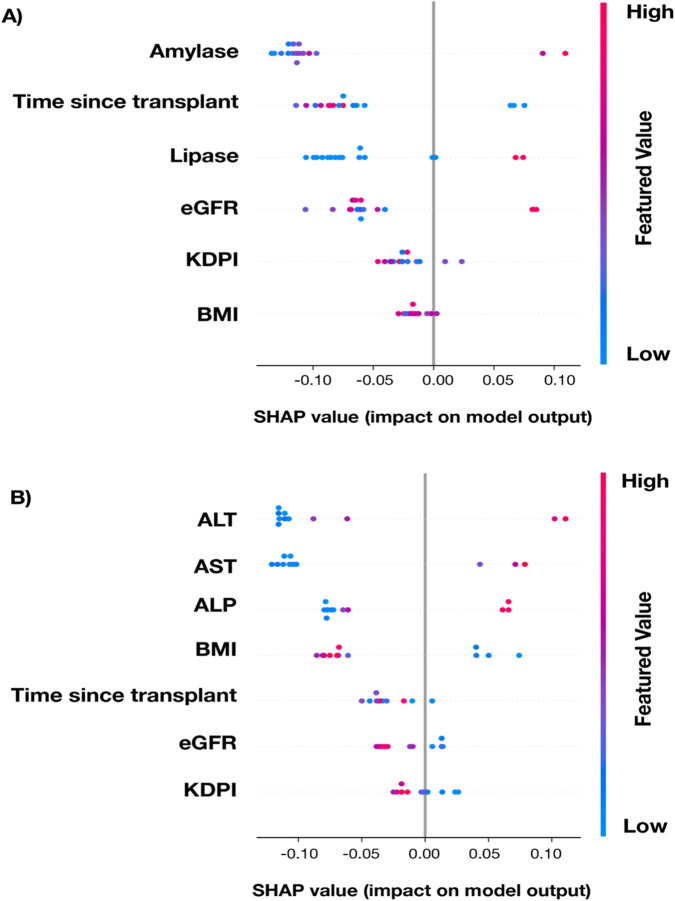
SHAP (SHapely Additive exPlanations) summary plot for a randomly-selected holdout testing subset of cases with complete data from the PKT **(A)** and LKT **(B)** transplant cohorts displaying the contribution of key variables to the random forest model’s prediction of increased-risk dd-cfDNA results. Each dot represents an individual patient. SHAP values on the x-axis indicate the variable’s impact on the model output (positive values increase likelihood of positive result, negative values decrease it). Features are ranked by overall importance from top to bottom. Color represents the variable value, with blue indicating low values and red indicating high values.

We sought to identify the clinical variables in MOT recipients that were associated with elevated dd-cfDNA levels using a random forest classifier with the PKT (Figure 4A) and LKT (Figure 4B) cohorts. The traditional markers for respective organ function were chosen as variables. In the PKT cohort, the most influential variables included amylase, time since transplant, and lipase. Shorter time post-transplant, higher amylase values, and higher lipase values were associated with higher likelihood of elevated dd-cfDNA (Figure 4A). Looking at the continuous relationship between dd-cfDNA and the laboratory markers in the PKT cohort, significant positive correlations were observed in the PKT cohort between dd-cfDNA% and amylase and lipase (p < 0.001, 0.006, respectively), as well as between DQS and amylase and lipase (p = 0.006, 0.027, respectively). eGFR did not show a significant association with dd-cfDNA in either the random forest model or using Spearman correlations (Supplementary Figure S3).

In the LKT cohort, the most influential variables in the random forest classifier were ALT, AST, and ALP. Higher liver enzyme profiles were associated with higher likelihood of elevated dd-cfDNA (Figure 4B). Across all dd-cfDNA levels in this cohort, significant positive correlations were observed between dd-cfDNA% and ALT and AST (p < 0.001 for both), and between DQS and ALT and AST (p < 0.001 for both). No significant correlations were found between dd-cfDNA levels and eGFR (Supplementary Figure S4).

As the model indicated a significant association between time post-transplant and the likelihood of elevated dd-cfDNA levels, we further assessed the relationship between dd-cfDNA and time post-transplant in the different MOT cohorts (Supplementary Figure S5). In the PKT and HKT cohorts, dd-cfDNA% and DQS (cp/mL) both decreased over time. Total cfDNA remained steady in the PKT cohort while the HKT cohort showed a dramatic decrease in the first 40 months post-transplant before stabilizing. Despite these fluctuations, dd-cfDNA levels in both cohorts remained below the validated thresholds for KT rejection. In the LKT cohort, dd-cfDNA% and DQS increased slightly over time, whereas the total cfDNA decreased slightly, before stabilizing.

## Discussion

This study represents the largest comprehensive characterization of dd-cfDNA (fraction and quantity) baseline levels in MOT recipients, addressing a gap in transplant biomarker research in this growing high-risk population. With characteristics comparable to the national SRTR registry, this cohort reflects real-world diversity and provides reference data for these populations. Key findings show that dd-cfDNA baselines vary by MOT combination and were generally aligned with known dd-cfDNA patterns from the major contributing organ: PKT and HKT had values comparable to KT recipients, while the higher dd-cfDNA levels in LKT recipients likely reflected the larger dd-cfDNA contribution from the liver transplants. Further, our data show associations between elevated dd-cfDNA and abnormal levels of traditional biomarkers used to test allograft functions indicating that dd-cfDNA levels reflect functional impairment of the respective transplanted organ (kidney, liver). Collectively, these data may help establish dd-cfDNA baseline levels for different MOT combinations, underscoring the potential for dd-cfDNA to serve as a non-invasive biomarker for rejection risk assessment in MOT recipients.

The release of dd-cfDNA from transplanted organs is influenced by several factors, including order of organ transplantation, organ size, organ vascularization, and cellular turnover rate [38]. The broad inclusion criteria include patients with both simultaneous and sequential transplants, allowing us to explore the impact of the order of organ transplant on dd-cfDNA levels. Our data suggests that the pancreas is the minor dd-cfDNA contributor in PKT recipients as the levels in SPKT were comparable to those of KT recipients. Similarly, the baseline dd-cfDNA levels in the HKT recipient’s cohort, including SHKT and KAHT, were comparable to those of KT recipients and significantly higher in SHKT than those of HT recipients, suggesting heart is the minor dd-cfDNA contributor relative to kidney. In contrast, dd-cfDNA levels were higher among the LKT cohort compared to KT recipients which is consistent with previous reports that LT recipients exhibit significantly higher dd-cfDNA levels than other solid organ transplants [35–37]. Given these data, it is not surprising that in this cohort, 2/3 PKT patients with kidney BPAR had dd-cfDNA above the 1.0% threshold established for KT rejection, as were 3/3 of the LKT patients with BPAR in the kidney. Of note, none of the three LKT patients with BPAR in the kidney had dd-cfDNA% above the exploratory 10% threshold proposed for liver transplant (LT) rejection; likely due to the lack of evidence of rejection in the liver of these patients. Taken together, these data suggest that the dd-cfDNA levels from the organ with the higher baseline may obscure rejections in the organ with the lower dd-cfDNA baseline, as seen in LKT and HKT (Supplementary Figure S2C). These findings underscore the importance of considering organ-specific dd-cfDNA levels when interpreting combined dd-cfDNA results in MOT populations and how it can be extrapolated from those established in single organ transplant recipients.

The clinical value of dd-cfDNA in MOT is further supported by its associations with abnormal levels in traditional biomarkers. In the PKT cohort, dd-cfDNA positivity was associated with increased serum amylase and lipase levels, suggesting that pancreas-derived injury signals correlate with higher dd-cfDNA release associated with rejection. Similarly, in the LKT cohort, strong associations between dd-cfDNA positivity and elevated liver enzymes (ALT, AST) are consistent with prior data suggesting that elevated dd-cfDNA levels in LT patients are associated with impaired liver function. These findings suggest that elevations in dd-cfDNA may serve as a sensitive indicator of allograft dysfunction across different transplanted organ types in MOT recipients.

In our study, the incidence of AR in our cohort (6.2%) was lower than that reported in similar MOT studies (24%) [39, 40] and (43%) [28]. This may reflect the large proportion of clinically stable patients enrolled in the study, which could be due to the relatively long post-transplant timeframe observed in this cohort. An added effect could be the immunoprotective role hypothesized in MOT recipients, which could explain the seemingly counterintuitive finding that rejection rates are lower in MOT than in single organ transplant. Previous studies have shown that patients with dual organ transplants involving the heart and/or liver, tend to exhibit greater graft stability compared to single transplants alone [41, 42]. While it was previously thought that only MOT patients with a liver allograft experienced this immunoprotective effect, data has now shown that other simultaneously-transplanted organs also have this immunologic benefit [8, 43, 44]. This effect was not observed in sequential transplants, indicating that shared antigenicity in simultaneous MOT may be required for immunoprotection to manifest [44]. In this study, the majority of participants were simultaneous MOT, which could have contributed to the lower incidence of AR via this ‘immunoprotective’ MOT mechanism.

A key challenge in MOT is identifying the graft undergoing rejection while managing immunosuppression to avoid toxicity. This becomes more complex when the transplanted organs are from different donors. The ability to discern the rejecting graft in these cases could be augmented by distinguishing between donor-specific cfDNA signatures, an approach proven feasible in a recent study of pregnant kidney recipients where fetal- and graft-derived cfDNA were successfully differentiated [45]. Analogous advances in noninvasive prenatal testing (NIPT), such as SNP-based assays that enable precise attribution of cfDNA to individual fetuses in twin pregnancies, demonstrate that identifying the tissue origin of circulating cfDNA is feasible [46]. While this approach would not allow for differentiation of organ-specific dd-cfDNA in simultaneous MOT where the two transplanted organs are from the same donor, other studies have revealed that the cfDNA methylation patterns can be used to identify the tissue-of-origin, independent of donor number or type [47].

This study has several limitations. First, biopsy data were not available for 72% of patients, with the designation of “non-rejecting” made based on clinical characteristics, and of those patients with a biopsy, very few patients had a biopsy from both organs. Second, the overall number of BPAR cases was insufficient to allow determination of a dd-cfDNA threshold for the clinical detection rejection in MOT recipients. Third, the study did not differentiate between dd-cfDNA contributions from multiple donors in MOT recipients, limiting the ability to precisely attribute dd-cfDNA elevations to a specific organ. Fourth, this analysis was cross-sectional in nature, limiting the assessment of the dynamic changes of dd-cfDNA over time.

In conclusion, this study is the largest of its kind, in which dd-cfDNA% and DQS baselines were characterized in pancreas-kidney, heart-kidney, and liver-kidney transplant recipients. This study provides foundational insights into how dd-cfDNA levels differ among and between different combinations of MOT, how dd-cfDNA levels relate to more traditional rejection biomarkers, and how these levels may be interpreted relative to each other. Future longitudinal studies focusing on MOT cohorts are needed to develop clinically valid assays to detect and define thresholds of organ-specific dd-cfDNA and improve rejections detection in these complex transplant scenarios.

## Data Availability

The original contributions presented in the study are included in the article/Supplementary Material, further inquiries can be directed to the corresponding authors.

## Glossary

ABMR: antibody-mediated rejection
ALT: alanine aminotransferase
ALP: alkaline phosphatase
AMBR: antibody-mediated rejection
AR: acute rejection
AST: aspartate aminotransferase
BMI: body mass index
BNP: B-type natriuretic peptide
BPAR: biopsy-proven acute rejection
cfDNA: cell-free DNA
CNI: calcineurin inhibitor
cp/mL: copies per milliliter
dd-cfDNA: donor-derived cell-free DNA
DQS: donor quantity score
DSA: donor-specific antibodies
eGFR: estimated glomerular filtration rate
HK: heart–kidney
HKT: heart–kidney transplant
HT: heart transplant
IQR: interquartile range
KAHT: kidney-after-heart transplant
KALT: kidney-after-liver transplant
KDPI: Kidney Donor Profile Index
KT: kidney transplant
LAKT: liver-after-kidney transplant
LK: liver–kidney
LKT: liver–kidney transplant
LT: liver transplant
MOT: multi-organ transplantation
MOTR: Multi-Organ Transplant Recipients study
NIPT: noninvasive prenatal testing
NT-proBNP: N-terminal pro–B-type natriuretic peptide
OPTN: Organ Procurement and Transplantation Network
PAK: pancreas-after-kidney transplant
PAKT: pancreas-after-kidney transplant
PKT: pancreas–kidney transplant
sCr: serum creatinine
SHKT: simultaneous heart–kidney transplant
SPKT: simultaneous pancreas–kidney transplant
SLKT: simultaneous liver–kidney transplant
SNP: single-nucleotide polymorphism
SRTR: Scientific Registry of Transplant Recipients
TCMR: T-cell–mediated rejection
