# Supplementary material for: Donor-Derived Cell-Free DNA in Pancreas-Kidney, Heart-Kidney, and Liver-Kidney Multiorgan Transplant Recipients (MOTR)
Source: Transpl Int. 2026 Jan 19;38:15823. doi: 10.3389/ti.2025.15823 (PMC12862255; doi:10.3389/ti.2025.15823)
Supplement: Supplementary file 1 [file DataSheet1.pdf]

Supplementary Materials

Figure S1: Total cfDNA approximation (cp/mL) in PKT (A), HKT (B), and LKT (C) cohorts**.** PKT: Kidney-Pancreas transplant, SPKT: Simultaneous Kidney-Pancreas transplant, PAKT: Pancreas-after-Kidney transplant, PK-RT: Pancreas-Kidney Repeat transplant, KT: Kidney transplant, HT: Heart transplant, HKT: Kidney-Heart transplant, KAHT: Kidney-after-Heart transplant, SLKT: Simultaneous kidney-liver transplant, KALT: Kidney-after-liver transplant, LAKT: Liver-after-kidney transplant, KL-RT: Kidney-liver Repeat transplant.

A)

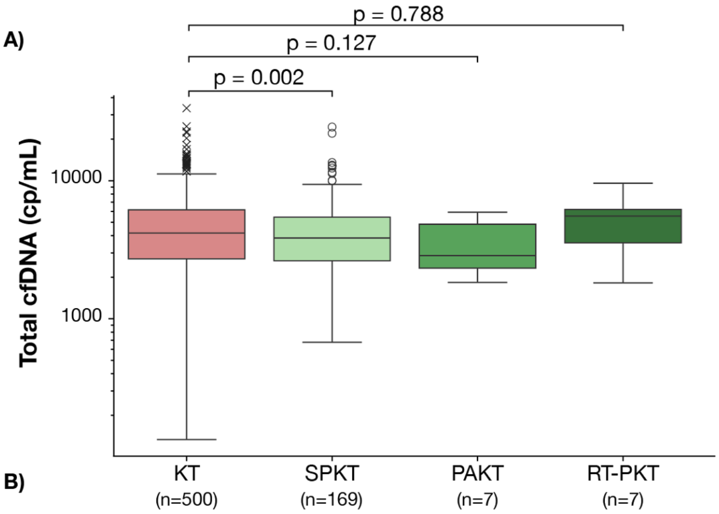
p = 0.788
p = 0.127
p = 0.002
Total cfDNA (cp/mL)
10000
1000
KT
(n=500)
SPKT
(n=169)
PAKT
(n=7)
RT-PKT
(n=7)

B)

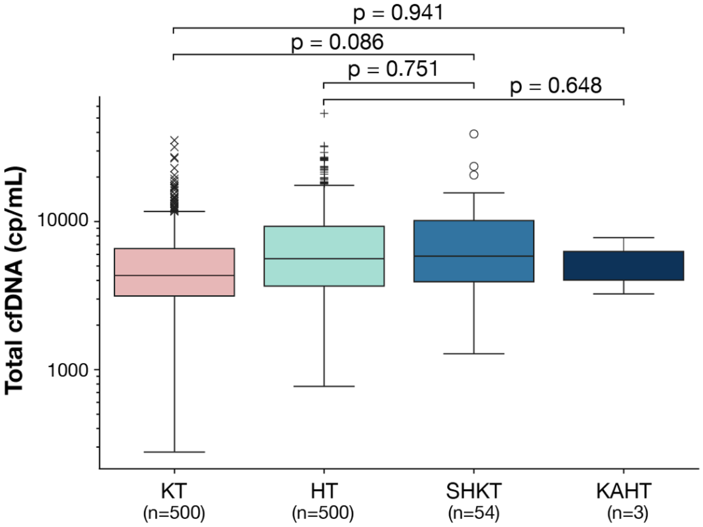
p = 0.941
p = 0.086
p = 0.751
p = 0.648
Total cfDNA (cp/mL)
10000
1000
KT
(n=500)
HT
(n=500)
SHKT
(n=54)
KAHT
(n=3)

C)

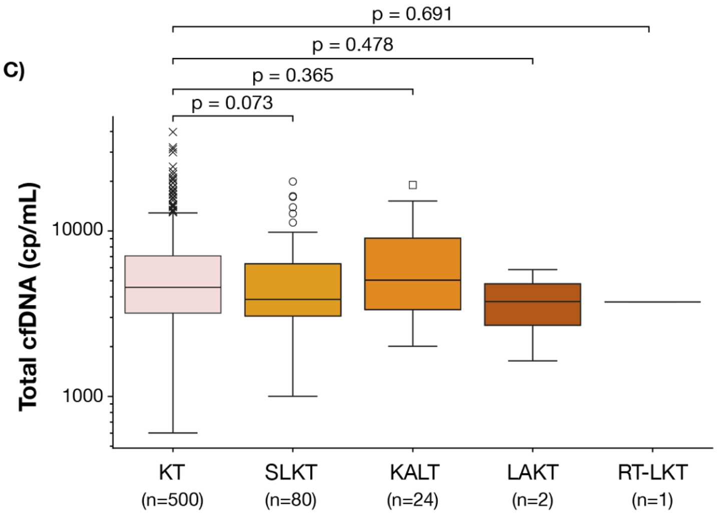
p = 0.691
p = 0.478
p = 0.365
p = 0.073
Total cfDNA (cp/mL)
10000
1000
KT
(n=500)
SLKT
(n=80)
KALT
(n=24)
LAKT
(n=2)
RT-LKT
(n=1)

Figure S2: A) Scatterplot of dd-cfDNA samples in the PKT (A) and HKT (B), and LKT (C) cohorts. A) The blue vertical and horizontal lines indicate the dd-cfDNA fraction and quantity thresholds used to assess kidney transplant rejection risk – 1.0% and 78 cp/mL, respectively. B) The red vertical and horizontal lines indicate the dd-cfDNA fraction and quantity thresholds used to assess heart transplant rejection risk – 0.26% and 18 cp/mL, respectively. C) The blue and red vertical lines indicate the dd-cfDNA fraction threshold used to assess kidney transplant and liver transplant rejection risk – 1.0% and 10.0%, respectively. The blue horizontal line represents the DQS threshold for kidney rejection (78 cp/mL).

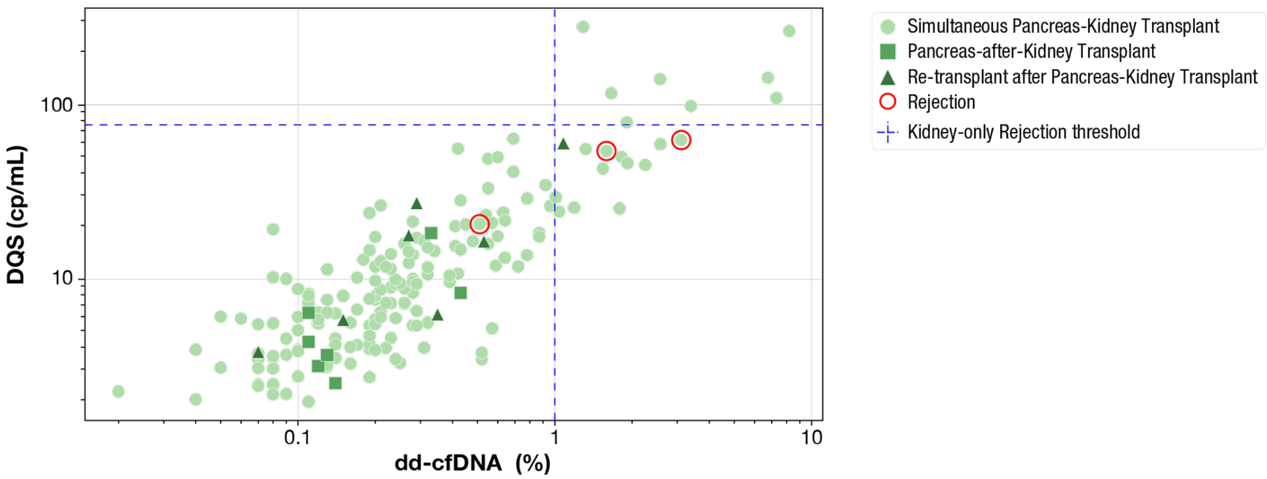

DQS (cp/mL)
100
10
0.1
1
10
dd-cfDNA (%)
Simultaneous Pancreas-Kidney Transplant
Pancreas-after-Kidney Transplant
Re-transplant after Pancreas-Kidney Transplant
Rejection
Kidney-only Rejection threshold

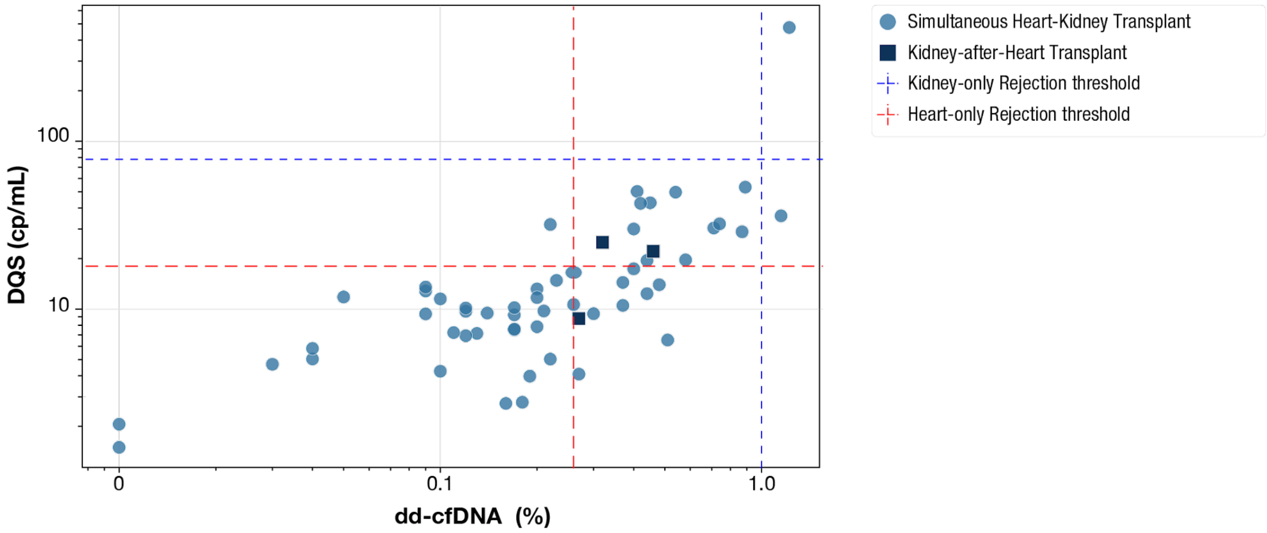

DQS (cp/mL)
100
10
0
0.1
1.0
dd-cfDNA (%)
Simultaneous Heart-Kidney Transplant
Kidney-after-Heart Transplant
Kidney-only Rejection threshold
Heart-only Rejection threshold

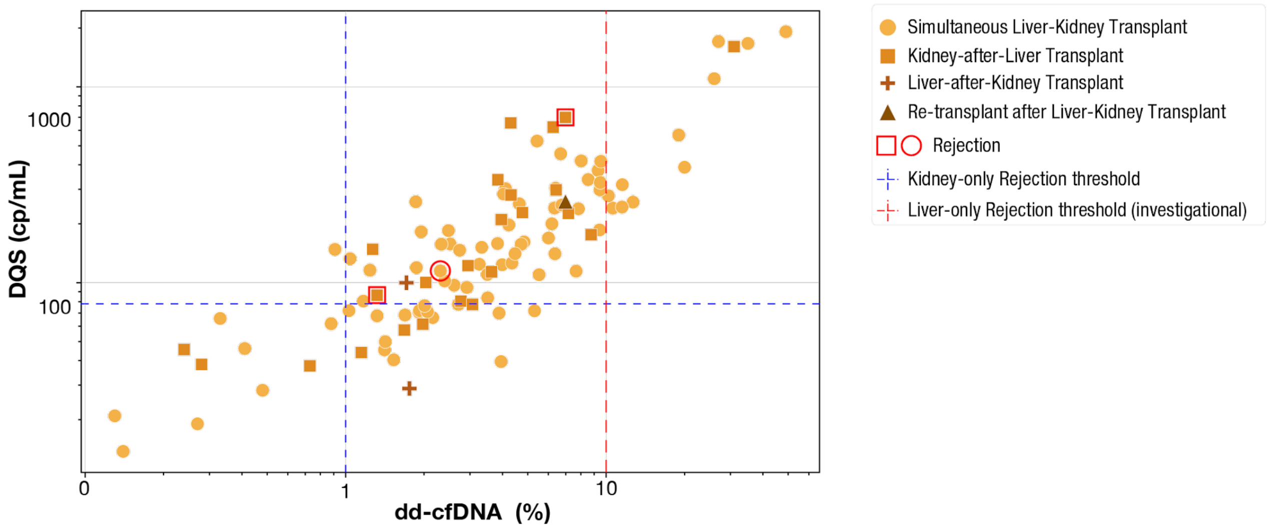

DQS (cp/mL)
1000
100
0
1
10
dd-cfDNA (%)
Simultaneous Liver-Kidney Transplant
Kidney-after-Liver Transplant
Liver-after-Kidney Transplant
Re-transplant after Liver-Kidney Transplant
Rejection
Kidney-only Rejection threshold
Liver-only Rejection threshold (investigational)

Figure S3: Correlations between amylase, lipase, and eGFR with dd-cfDNA fraction (%) and DQS (cp/mL) in the PKT cohort.

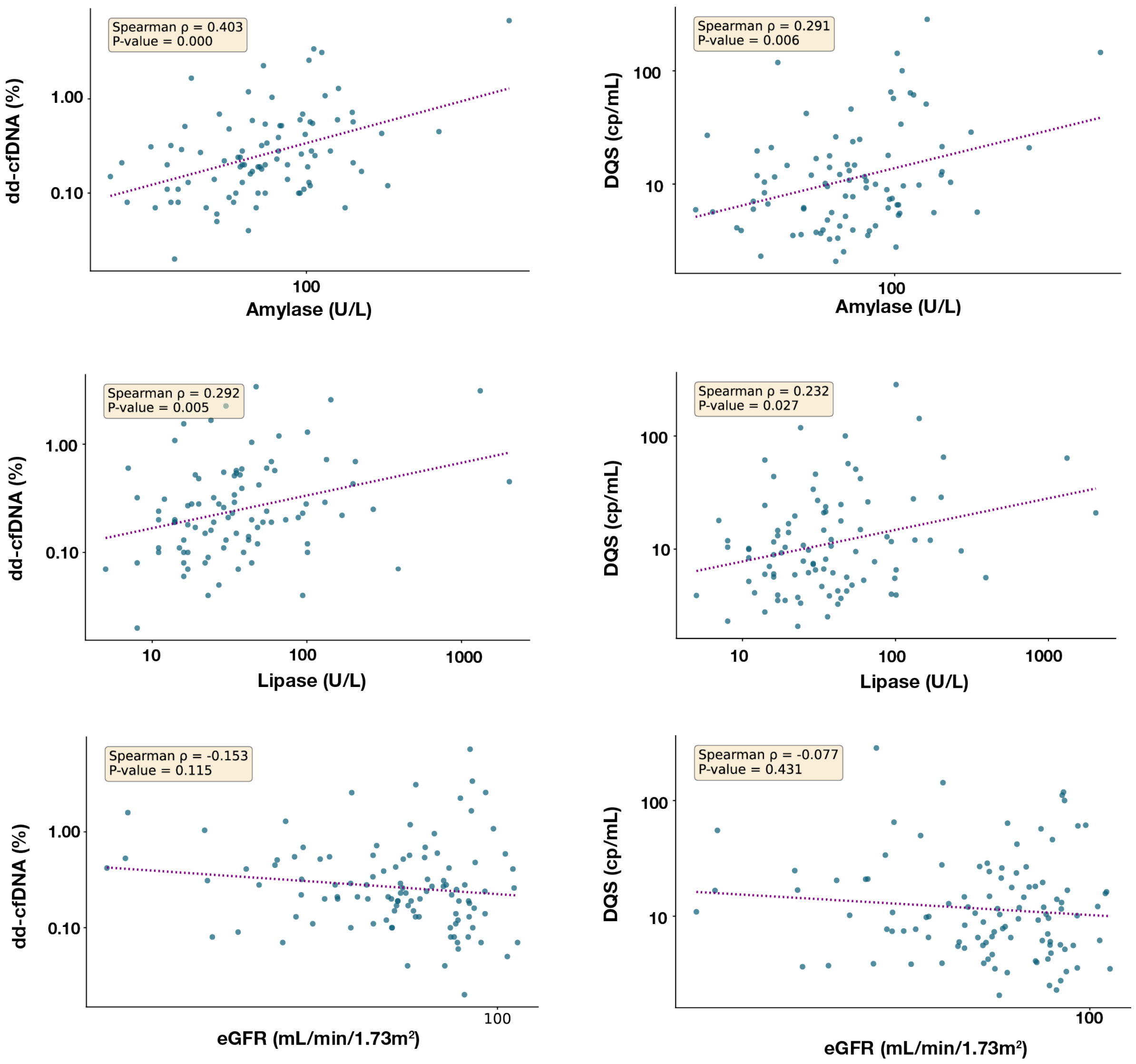

Figure S4: Correlations between ALP, ALT, AST, and eGFR with dd-cfDNA fraction (%) and DQS (cp/mL) in the LKT cohort.

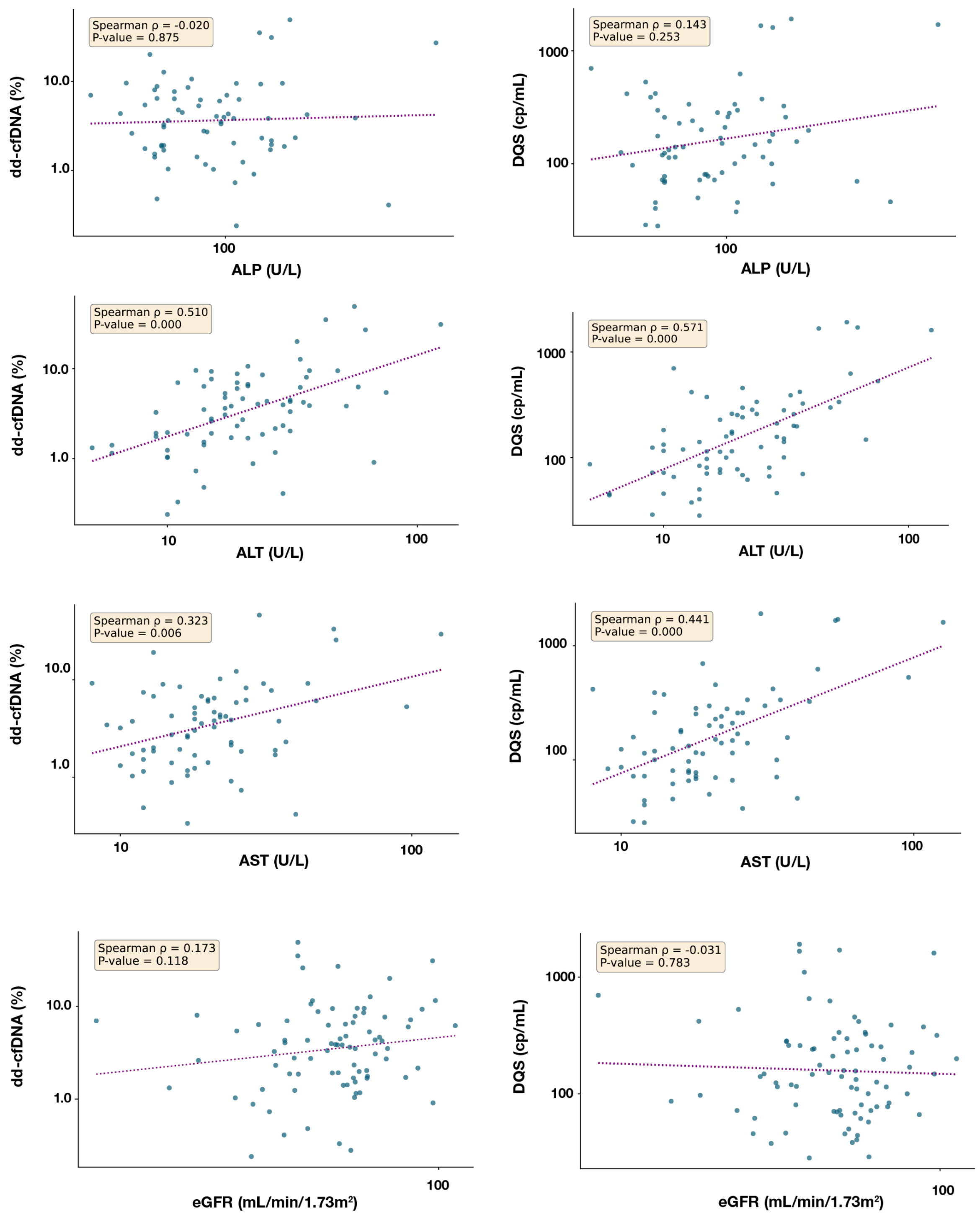

Figure S5: Association between time post-transplant and dd-cfDNA fraction (%), DQS (cp/mL) and total cfDNA in the PKT, HKT, and LKT cohorts**.** All axes are log-scaled, and the Lowess regression line represents the overall trend.

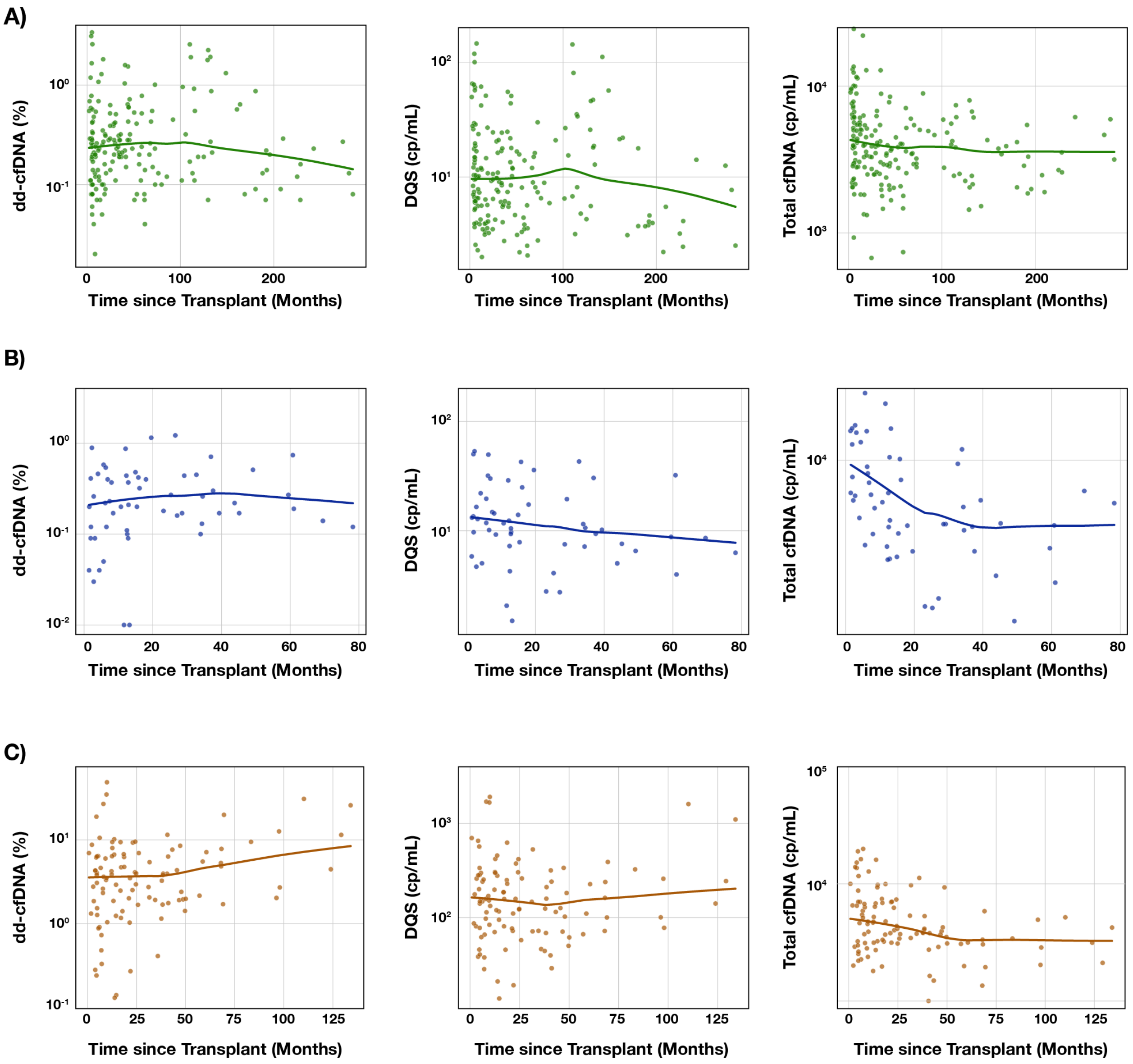

Table S1: A list of the MOTR study investigators with their respective centers and the IRB approval protocols numbers.

	Site PI	Site name	IRB	Central IRB #	Local IRB #
1	Gaurav Gupta	Virginia Commonwealth University, Richmond, VA, USA	Central	WCG IRB Study #: 1295889 IRB Tracking #: 20200682	-
2	Nicolae Leca	University of Washington, Seattle, WA	Central	WCG IRB Study #: 1284159 IRB Tracking #: 20200682	-
3	Ziad Zaky	Cleveland Clinic, Cleveland, OH	Local	-	Cleveland Clinic IRB#: 10-720
4	Alp Sener	London Health, Ontorio, Canada	Local	-	Western University Health Sciences Research Ethics Board (HSREB) Project ID#: 116387
5	Milagros Samanaiego-Picota	Henry Ford, Detroit, MI	Local	-	Henry Ford Health Systems Institutional Review Board #: 14100
6	David Wojciechowski	UT Southwestern, Dallas, Tx	Central	WCG IRB Study #: 1295457 IRB Tracking #: 20200682	-
7	Robert Cannon	University of Alabama, Birmingham, AL	Central	WCG IRB Study #: 1305853 IRB Tracking #: 20200682	-
8	Karthik Ramanathan	University of Minnesota, Minneapolis, MN	Central	WCG IRB Study #: 1305977 IRB Tracking #: 20200682	-
9	Ty Dunn	University of Pennsylvania, Philadelphia, PA	Local	-	University of Pennsylvania Institutional Review Board, Protocol #: 844937
10	Todd Pesavento	Ohio State University, Columbus, OH	Central	WCG IRB Study #: 1296401 IRB Tracking #: 20200682	-
11	Richard Ugarte	University of Maryland, Baltimore, MD	Central	WCG IRB Study #: 1296962 IRB Tracking #:	-

	Site PI	Site name	IRB	Central IRB #	Local IRB #
				20200682	
**12**	Kasi McCune	Columbia University, New York, NY	Local	-	Columbia Research Human Protection Program, Protocol #: IRB-AAA-T2004
**13**	Sanjeev Akkina	Loyola University Medical Center, Maywood, IL	Local	-	Loyola University Chicago, Health Sciences Division, Institutional Review Board for the Protection of Human Subjects, LU #: 213705
**14**	Teresa Rice	Medical University of South Carolina, Charleston, SC	Central	WCG IRB Study #: 1303517 IRB Tracking #: 20200682	-
**15**	Jon Odorico	University of Wisconsin, Madison, WI	Central	WCG IRB Study #: 1305266 IRB Tracking #: 20200682	-
**16**	Timothy Gong	Baylor University, Dallas, Tx	Local	-	Baylor Scott & White Research Institute Institutional Review Board, IRB No: 021-008
**17**	Jason Vanatta	Methodist Memphis- James Eason Transplant Center, Memphis, TN	Central	WCG IRB Study #: 1304003 IRB Tracking #: 20200682	-
**18**	Swati Rao	University of Virginia, Charlottesville, VA	Central	WCG IRB Study #: 1320467 IRB Tracking #: 20200682	-

Table S2: Comparison of our study cohort and sub-cohorts to contemporaneous cohorts

	PKT cohort (n = 183)	Contemporaneous SRTR PKT recipients (n = 27358)	HKT cohort (n=57)	Contemporaneous SRTR HKT recipients (n = 3145)	LKT cohort (n =107)	Contemporaneous SRTR LKT recipients (n = 14411)	Pairwise p-values** PKT-HKT PKT-LKT HKT-LKT
**Age at most recent transplant (years)**	42.5 (37.5 - 49.8)	42 (35 - 48)	57.4 (48.4 - 63.2)	59 (50 - 65)	58. 5 (53.0 - 64.3)	58 (50 - 63)	0.14<br>0.21<br>0.17
**Biological Sex**							0.46<br>0.93<br>0.41
**Male**	115 (62.8%)	16385 (59.89%)	45 (79.0%)	2440 (77.6%)	64 (59.8%)	9242 (64.13%)	
**Female**	68 (37.2%)	10973 (40.11%)	12 (21.1%)	705 (22.4%)	43 (40.2%)	5169 (35.9%)	
**BMI**[1] **(kg/m²)**	27.2 (24.1 - 31.8)	24.7 (22.2 - 27.7)	28.9 (26.0 - 32.9)	26.3 (23.1 - 29.9)	29.0 (26.0 - 33.4)	26.5 (23.1 - 30.6)	1.2e-13<br>5.4e-5<br>3.6e-6
**Race**							0.0<br>6.8e-9<br>0.002[2]
**White**	82 (44.82%)	22137 (80.92%)	18 (31.58%)	2200 (69.95%)	79 (73.83%)	11798 (81.87%)	
**Black or African American**	44 (24.04%)	4503 (16.46%)	32 (56.14%)	787 (25.02%)	20 (18.69%)	1832 (12.71%)	
**Other**	54 (29.51%)	218 (0.80%)	2 (3.51%)	24 (0.76%)	5 (4.67%)	171 (1.19%)	
**Asian**	3 (1.64%)	500 (1.83%)	5 (8.77%)	134 (4.26%)	3 (2.80%)	610 (4.23%)	
**Ethnicity**							0.442<br>0.599<br>0.054[3]
**Not Hispanic or Latino**	116 (63.39%)	24690 (90.25%)	53 (92.98%)	2887 (91.80%)	94 (87.85%)	12073 (83.78%)	
**Hispanic or Latino**	16 (8.74%)	2668 (9.75%)	3 (5.26%)	258 (8.20%)	9 (8.41%)	2338 (16.22%)	
**Not Reported or Unknown**	51 (27.87%)	0 (0%)	1 (1.75%)	0 (0%)	4 (3.74%)	0 (0%)	
**Repeat transplant status**							1.6e-5<br>0.199<br>0.002
**Single**	176 (96.2%)	23051 (84.3%)	55 (96.5%)	2850 (90.6%)	104 (97.2%)	12499 (86.7%)	
**Repeat**	7 (3.83%)	4307 (15.7%)	2 (3.5%)	295 (9.4%)	3 (2.8%)	1912 (13.3%)	

BMI: Body mass index, PKT: pancreas kidney transplant, HKT: heart kidney transplant, LKT: liver kidney transplant, SRTR: The Scientific Registry of Transplant Recipients.

---

1. In the SRTR dataset, apparent data entry errors were observed in BMI values (e.g., values below 1 or above 10,000 kg/m²). To address this, published guidelines were used to define a valid BMI range of 12 to 60 for inclusion in the analysis [1].
2. The observed association is likely influenced by missing ethnicity data in the MOTR cohort. In contrast, the SRTR dataset does not contain missing values for ethnicity. As a result, p-values for ethnicity comparisons were calculated only among individuals with known ethnicity, and unknowns in the MOTR cohort were excluded from these specific analyses.

3. The p-values were calculated based solely on individuals with known ethnicity, as the SRTR cohorts did not contain missing ethnicity data.

References

1. Naik AS, Cibrik DM, Sakhuja A, et al. Temporal trends, center-level variation, and the impact of prevalent state obesity rates on acceptance of obese living kidney donors. *Am J Transplant*. Mar 2018;18(3):642-649. doi:10.1111/ajt.14519

Capsule Sentence:

This multicenter study defines baseline dd-cfDNA levels across pancreas–kidney, heart–kidney, and liver–kidney multiorgan transplant recipients. Findings demonstrate organ-specific physiologic effects on dd-cfDNA and establish foundational reference data to support future use of dd-cfDNA for noninvasive rejection assessment in multiorgan transplantation.
